# The prevalence of vancomycin-resistant *Staphylococcus aureus* in Ethiopia: a systematic review and meta-analysis

**DOI:** 10.1186/s13756-023-01291-3

**Published:** 2023-08-30

**Authors:** Melaku Ashagrie Belete, Alemu Gedefie, Ermiyas Alemayehu, Habtu Debash, Ousman Mohammed, Daniel Gebretsadik, Hussen Ebrahim, Mihret Tilahun

**Affiliations:** Department of Medical Laboratory Sciences, College of Medicine and Health Sciences, Wollo University, Dessie, Ethiopia

**Keywords:** Vancomycin-resistant *Staphylococcus aureus*, Systematic review, meta-analysis, Ethiopia

## Abstract

**Introduction:**

Vancomycin-resistant *Staphylococcus aureus*, identified as a “high priority antibiotic-resistant pathogen” by the World Health Organization, poses a significant threat to human health. This systematic review and meta-analysis aimed to estimate the pooled prevalence of vancomycin-resistant *Staphylococcus aureus* in Ethiopia.

**Methods:**

This systematic review and meta-analysis was reported in accordance with the Preferred Reporting Items for Systematic Reviews and Meta-Analyses guidelines. Studies that reported VRSA prevalence due to infection or carriage from human clinical specimens were extensively searched in bibliographic databases and grey literatures using entry terms and combination key words. Electronic databases like PubMed, Google Scholar, Wiley Online Library, African Journal Online, Scopus, Science Direct, Embase, and ResearchGate were used to find relevant articles. In addition, the Joanna Briggs Institute quality appraisal tool was used to assess the quality of the included studies. Stata version 14 software was used for statistical analysis. Forest plots using the random-effect model were used to compute the overall pooled prevalence of VRSA and for the subgroup analysis. Heterogeneity was assessed using Cochrane chi-square (I^2^) statistics. After publication bias was assessed using a funnel plot and Egger’s test, trim & fill analysis was carried out. Furthermore, sensitivity analysis was done to assess the impact of a single study on pooled effect size.

**Results:**

Of the 735 studies identified, 31 studies that fulfilled the eligibility criteria were included for meta-analysis consisted of 14,966 study participants and 2,348 *S. aureus* isolates. The overall pooled prevalence of VRSA was 14.52% (95% CI: 11.59, 17.44). Significantly high level of heterogeneity was observed among studies (I^2^ = 93.0%, p < 0.001). The region-based subgroup analysis depicted highest pooled prevalence of 47.74% (95% CI: 17.79, 77.69) in Sidama region, followed by 14.82% (95% CI: 8.68, 19.88) in Amhara region, while Oromia region had the least pooled prevalence 8.07% (95% CI: 4.09, 12.06). The subgroup analysis based on AST methods depicted a significant variation in pooled prevalence of VRSA (6.3% (95% CI: 3.14, 9.43) for MIC-based methods, and 18.4% (95% CI: 14.03, 22.79) for disk diffusion AST method) which clearly showed that disk diffusion AST method overestimates the pooled VRSA prevalence. The total number of *S. aureus* isolates was found to be the responsible variable for the existence of heterogeneity among studies (p = 0.033).

**Conclusion:**

This study showed an alarmingly high pooled prevalence of VRSA necessitating routine screening, appropriate antibiotic usage, and robust infection prevention measures to manage MRSA infections and control the emergence of drug resistance. Furthermore, mainly attributable to the overestimation of VRSA burden while using disk diffusion method, there is an urgent need to improve the methods to determine vancomycin resistance in Ethiopia and incorporate MIC-based VRSA detection methods in routine clinical laboratory tests, and efforts should be directed at improving it nationally.

**Trial Registration:**

PROSPERO registration identification number: CRD42023422043.

**Supplementary Information:**

The online version contains supplementary material available at 10.1186/s13756-023-01291-3.

## Introduction

Bacterial multidrug resistance has emerged as a global threat, and continues to pose a significant challenge to medicine and healthcare systems worldwide [1]. There has been a devastating report of about 5 million deaths globally associated with bacterial antimicrobial resistance (AMR) only in the year 2019, of which sub-Saharan Africa bear the highest burden, with 27.3 deaths per 100,000 attributable to AMR. Surprisingly, it is also predicted that AMR will possibly kill 10 million people annually by 2050, while tumbling the global economy by $100 trillion [2].

*Staphylococcus aureus* (*S. aureus*), which is a Gram-positive coccus responsible for various human infections, ranging from skin and soft tissue infections to life-threatening systemic diseases as an opportunistic, nosocomial and community-acquired pathogen [3]. Over the years, *S. aureus* has developed various drug resistance mechanisms, which make it difficult to treat with conventional antibiotics, including βeta-lactamase production, methicillin resistance (MRSA), vancomycin resistance (VRSA), macrolide, aminoglycoside and quinolone resistances, and biofilm formation [4]. Highly drug resistant *S. aureus* including MRSA have been effectively treated with vancomycin as a first line drug since 1980s [5, 6], and vancomycin has been used as a last resort antibiotic for the management of severe infections due to MRSA and other MDR Gram-positive pathogens [7]. However, *S. aureus* isolates resistant to vancomycin have emerged in the past two decades, and are now becoming a major cause of morbidity and mortality worldwide [7, 8], with the first VRSA being reported in 1997 from Japan [9].

The World Health Organization has recently listed VRSA as a “high priority antibiotic-resistant pathogens” [10] due to its significant impact on public health. Vancomycin resistance in *S. aureus* (MIC ≥ 16 µg/ml) is mainly conferred by *vanA* operon encoded on transposon Tn1546, and other *van* gene clusters including *vanB, vanC, vanD, vanF, vanE, vanG vanI, vanL, vanM* and *vanN* phenotypes [11, 12]. These genetic elements alter the cell wall structure, preventing vancomycin from effectively inhibiting cell wall synthesis [13, 14]. Primarily due to their evidently decreased permeability and altered cell wall, VRSA strains are immensely multidrug resistant against various antibacterial agents currently in use [6].

Recently, published systematic review and meta-analysis articles assessed the epidemiology of VRSA globally and revealed the prevalence based on diverse years and regions [15, 16]. Despite the reported morbidity rates of VRSA were relatively low in developed countries, the burden is still high in developing countries such as Africa. Thus, comprehensive countrywide studies are critical in low-income countries to reflect the real burden of VRSA nationally and devise control strategies.

In Ethiopia, there is a rapidly increasing bacterial antimicrobial resistance to the routinely used antibacterial drugs as depicted by a recent systematic review [17]. Thus, epidemiological studies and evidence-based practices are of paramount significance for developing effective prevention and control strategies and improving healthcare services. Although upsurging rates of VRSA are nowadays being reported in different parts of the world, there is no national pooled data in Ethiopia. This study is the first systematic review and meta-analysis to report the national burden of VRSA in Ethiopia; and it aimed to summarize the findings of local studies reporting VRSA infection or colonization, and estimate the pooled prevalence of VRSA in Ethiopia.

## Methods

### Guidelines and protocol registration

This systematic review and meta-analysis was reported in accordance with the Preferred Reporting Items for Systematic Reviews and Meta-Analyses guidelines (PRISMA) [18]. The protocol for this review was originally registered in the International Prospective Register of Systematic Reviews (PROSPERO) database with registration identification number of CRD42023422043.

### Search strategy and selection of studies

A comprehensive and systematic literature searches were carried out to retrieve studies reporting the prevalence of vancomycin-resistant *Staphylococcus aureus* (VRSA) in Ethiopia from different electronic bibliographic databases including PubMed/ Medline, Google Scholar, Wiley Online Library, African Journal Online, Scopus, Science Direct, Embase, and ResearchGate. Furthermore, grey literatures and university repositories were screened, and a direct Google search was carried out using the reference lists of the included studies to incorporate further relevant studies that was missed during electronic database searches. The search was conducted from May 1 to 20, 2023. Studies that were published/reported until April 30, 2023 and fulfilled the eligibility criteria were included.

A thorough searching strategy was deployed using the condition, context, population, and outcome of interest (CoCoPop) formulating questions, and all potentially eligible studies were accessed by using the following Medical Subject Headings (MeSH) terms and combination key words: “Prevalence”, “epidemiology”, “burden”, “*Staphylococcus aureus*”, “*S. aureus*”, “vancomycin-resistant *Staphylococcus aureus*”, “vancomycin-resistant *S. aureus*”, “VRSA” and “Ethiopia”. In the advanced searching databases, the abovementioned search terms were linked using Boolean operators (“OR” and “AND”) as necessary. Moreover, the bibliographies of all included studies were checked for additional articles and authors were contacted to receive any missing papers. Search results were consolidated into Endnote 20 software (Clarivate Analytics USA) and duplicates were removed. Three independent reviewers (MAB, AG and EA) identified the articles from databases and other sources. Duplicates were removed and four independent reviewers (HD, MT, OM, HE) screened the titles and abstracts of all retrieved studies, and were double-checked by a third reviewer (AG). The full texts of potentially eligible studies were then evaluated in detail against the inclusion criteria by two reviewers (MAB and EA), double-checked by a third reviewer (AG), and added to the extraction collection. Any disagreements among reviewers throughout each stage of screening were unraveled through discussion or with the intruding of a third reviewer (AG). Detailed article search strategies and search lines were indicated in Supplementary file 1.

### Eligibility criteria

Original studies published in peer-reviewed journals or grey literature, articles published in English language, studies that reported prevalence of vancomycin-resistant *Staphylococcus aureus* among clinical specimens recovered from any human study participants which encompassed infection or carriage, studies that detected vancomycin-resistance using phenotypic or genotypic methods, laboratory-based observational (e.g. cross-sectional) studies conducted in Ethiopia from January 1, 2000 to April 30, 2023, addressing the research question, and studies involving human (infected individuals or asymptomatic carriers) were included.

Studies were excluded if they were done from non-human sources. Qualitative studies, reviews, commentaries, letters to the editor, author replies, and studies that did not include quantitative data on the prevalence of VRSA were excluded. Furthermore, studies with duplicate data or overlapping articles, studies with outcomes of interest were missing or vague, and studies with a small number of *S. aureus* isolates (less than 10) were excluded.

### Outcome variables

The outcome variable for this study is the pooled prevalence of VRSA (infection and colonization) among Ethiopian populations. We included studies that reported the prevalence of VRSA among clinical specimens recovered from any human study participants, which encompassed both infection or carriage. In this study, an outcome of “infection” is defined as a form of diseases with suspected *S. aureus* aetiology by clinicians, while an outcome of “carriage” is defined as colonization of human with *S. aureus* as asymptomatic carrier, both of which are explained with detection of VRSA from human clinical specimens using phenotypic or genotypic methods.

### Quality assessment

Three authors (MAB, DG and EA) critically assessed the methodological and finding quality of the eligible studies using the Joanna Briggs Institute (JBI) quality appraisal tool for prevalence studies [19]. Using the critical appraisal checklist, studies with an average quality score of 50% or higher were deemed to be of good quality and hence included for analysis (Supplementary file 2). Studies were assessed using title, abstract and full text screening.

### Data extraction

Essential data from the eligible studies were extracted onto an excel spreadsheet by three reviewers (MAB, EA and AG). The extracted data include author (s) name, publication year, region, study area, study period, study design, study population, specimen types, antimicrobial susceptibility testing (AST) method, sample size, number of *S. aureus* isolates, number of VRSA isolates and prevalence of VRSA. The three reviewers thoroughly cross-checked their extraction outputs, and disagreements were resolved by discussion, data cross-checking and validation.

### Statistical data analysis

Data analysis was conducted using Stata version 14.0 software (Stata Corp., College Station, TX). We used logit transformation in our analysis to pool proportions. A random-effect model of DerSimonian and Laird analysis was used to estimate the pooled prevalence of VRSA [20]. The Cochran’s Q test and I^2^ statistics were used to quantify and assess the presence of heterogeneity between studies [21]. The p-value of < 0.05 for I^2^ statistics was used to determine the presence of heterogeneity. A predefined subgroup analysis was performed based on publication year, region, city, study design and AST method. Moreover, sensitivity analysis was carried out to assess the effect of a single study on the overall pooled estimate using a leave-one-out approach. Meta-regression was also used to further explore the potential sources of heterogeneity among the included studies by examining the relationship between study characteristics (such as publication year, sample size, or number of *S. aureus*) and the observed variations in the prevalence of VRSA, allowing for a more comprehensive understanding of the factors contributing to the heterogeneity. Publication bias was evaluated using inspection of funnel plot symmetry and Egger’s test statistics [22, 23]. The Trim-and-Fill analysis was then used in asymmetrical funnel plots to incorporate missing studies and provide an indication of the reliability of the estimate in relation to publication bias. The findings were presented using a pooled prevalence with a 95% CI, corresponding p-value and forest plots.

## Results

### Selection of studies

**Fig. 1:**
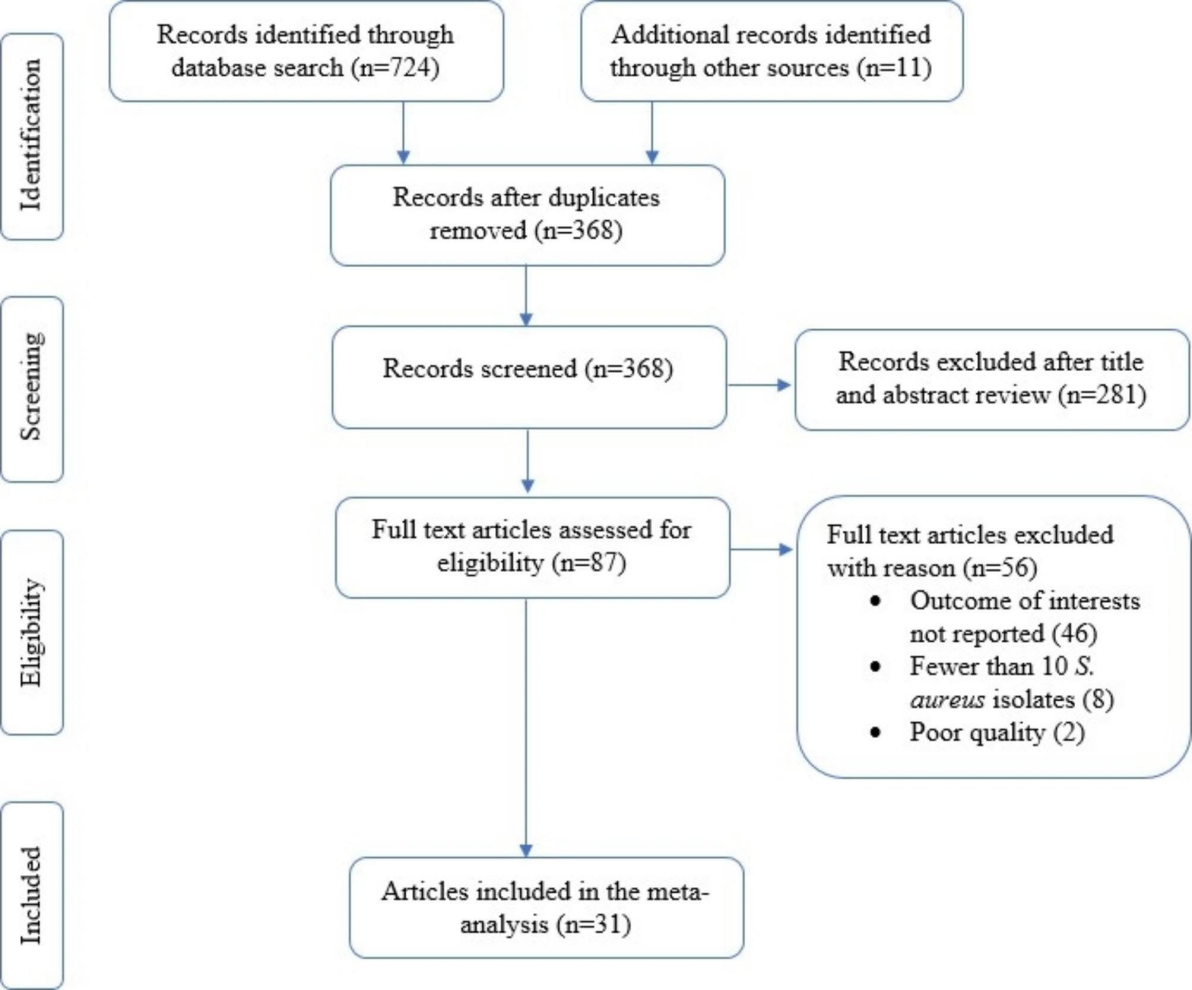
PRISMA flow diagram illustrating the process of selecting eligible studies for the systematic review and meta-analysis

A total of 735 studies were retrieved from database searches and other sources, from which 367 were removed due to duplication. The remaining 368 articles were screened based on title and abstract review, and 281 were removed. Finally, a total of 87 articles were thoroughly evaluated against the eligibility criteria, and only 31 were found to be potentially eligible for inclusion in the systematic review and meta-analysis (Fig. 1).

### Study characteristics

This systematic review and meta-analysis included a total of 31 original articles from different regions of Ethiopia. All the included studies had a quality score of greater than 75%. The overall number of participants in all studies included in the analysis was 14,966, with 315 VRSA isolates investigated from a total of 2,348 *S. aureus* isolates. Majority of the included (83.9%) deployed cross-sectional study design while the rest employed retrospective study design (Table 1).

**Table 1:** Characteristics of included studies

Authors & pub. year	Region	Study area	Study period	Study design	Study participants	Type of specimen	Outcome	AST method	Sample size	Prev. of S. aureus n (%)	Prev. of VRSA n (%)
Alebachew et al., 2012 [24]	Central	Addis Ababa	March to May 2011	Cross-sectional	Burn patients	Wound swab	Infection	Disk diffusion	114	66 (57.8)	4 (6.1)
Tadesse S., 2014 [25]	Central	Addis Ababa	December 2013 to June 2014	Cross-sectional	Inpatient and outpatients, post-surgical infection, otitis media suspects	Wound swab, ear swab, nasal swab	Infection	Disk diffusion	94	54 (57.4)	22 (40.7)
Negussie et al., 2015 [26]	Central	Addis Ababa	October 2011 to February 2012	Cross-sectional	Septicemia suspected children (≤ 12 years)	Blood	Infection	Disk diffusion	201	13 (23.2)	2 (15.4)
Dilnessa & Bitew, 2016 [27]	Central	Addis Ababa	September 2013 to April 2014	Cross-sectional	In and out patients	Nasal swab, pus/abscess, ear discharge, blood, throat swab, eye swab, vaginal discharge, urethral discharge, urine, stool, sputum, CSF, body fluids	Infection	E-test/MIC	1360	194 (14.3)	10 (5.2)
Dilnessa & Bitew, 2016 [28]	Central	Addis Ababa	September 2013 to August 2014	Cross-sectional	Postoperative and burn patients	Wound/pus swab	Infection	Dilution/MIC	378	179 (47.4)	10 (5.6)
Atlaw et al., 2022 [29]	Central	Addis Ababa	November 2020 to May 2021	Cross-sectional	Diabetic foot ulcer patients	Ulcer swab, pus aspirate	Infection	Disk diffusion	130	32 (25.2)	12 (37.5)
Gebremariam et al., 2022 [30]	Central	Addis Ababa	January to April 2018	Cross-sectional	All patients	Wound, blood, urine, ear swab, nasal swab, body fluid, eye swab, CSF, semen, urogenital swab	Infection	Dilution/MIC	792	54 (54)	1 (1.8)
Kahsay et al., 2014 [31]	Amhara	Debre Markos	December 2011 to March 2012	Cross-sectional	Patients with surgical site infections	Wound swab	Infection	Disk diffusion	184	73 (39.7)	3 (4.1)
Shibabaw et al., 2014 [32]	Amhara	Dessie	November 2010 to March 2011	Cross-sectional	Healthcare workers	Nasal swab	Carriage	Disk diffusion	118	34 (28.8)	10 (29.4)
Denboba et al., 2016 [33]	Amhara	Dessie	September 2001 to September 2011	Retrospective	Middle ear bacterial infection suspects	Ear discharge swab	Infection	Disk diffusion	1225	77 (6.3)	32 (41.6)
Abebe M et al., 2019 [34]	Amhara	Debre Markos	November 2013 to February 2017	Retrospective	In and out patients	Urine, stool, blood, ear discharge, wound, vaginal discharge, urethral discharge, CSF	Infection	Dilution/MIC	514	41 (17.1)	5 (12.2)
Gobena A, 2019 [35]	Amhara	Bahir Dar	January to December 2018	Cross-sectional	Pediatric patients	Blood	Infection	Disk diffusion	910	86 (9.4)	15 (17.4)
Abosse et al., 2020 [36]	Amhara	Bahir Dar	February to June 2019	Cross-sectional	Patients with surgical wound infections	Wound/pus swab	Infection	Disk diffusion	165	31 (26.9)	3 (9.7)
Tefera et al., 2021 [37]	Amhara	Debre Markos	February to April 2020	Cross-sectional	Inpatients	Wound swab	Infection	E-test/MIC	242	71 (29.3)	21 (29.6)
Jemal et al., 2021 [38]	Amhara	Gondar	January 2010 to December 2020	Retrospective	Neonatal sepsis suspects	Blood	Infection	Disk diffusion	1854	118 (22)	8 (6.8)
Getaneh et al., 2021 [39]	Amhara	Gondar	January 2013 to December 2018	Retrospective	Ear infection suspects	Ear discharge	Infection	Disk diffusion	369	75 (27.9)	4 (5.3)
Abebe W et al., 2021 [40]	Amhara	Gondar	January 2012 to December 2018	Retrospective	Blood stream infection suspects	Blood	Infection	Disk diffusion	2404	215 (8.9)	10 (4.6)
Abrha et al., 2011 [41]	Oromia	Jimma	October 2009 to May 2010	Cross-sectional	Severely malnourished children (below 14 years)	Blood	Carriage	Disk diffusion	170	10 (28.6)	3 (30)
Wubshet et al., 2012 [42]	Oromia	Jimma	February to April 2008	Cross-sectional	Inpatient and outpatients	Wound swab, nasal swab	Infection	Disk diffusion	323	81 (25.1)	1 (1.2)
Kejela & Bacha, 2013 [43]	Oromia	Jimma	December 2010 to June, 2011	Cross-sectional	Primary school children and prisoners	Nasal swab	Carriage	Disk diffusion	354	169 (47.7)	5 (3)
Godebo et al., 2013 [44]	Oromia	Jimma	June to December 2011	Cross-sectional	Inpatient and outpatient	Wound swab	Infection	Disk diffusion	322	73 (22.7)	12 (16.4)
Tesfaye et al., 2013 [45]	Oromia	Jimma	January to June 2012	Cross-sectional	External ocular infection suspects	Eyelid, conjunctiva, cornea and conjunctival swabs	Infection	Disk diffusion	198	42 (28.4)	7 (16.7)
Beyene et al., 2019 [46]	Oromia	Jimma	February to May 2017	Cross-sectional	Food handlers in hotels	Nasopharyngeal swab, hand swab	Carriage	Disk diffusion	300	86 (28.7)	6 (7)
Sorsa et al., 2019 [47]	Oromia	Asella	April 2016 to May 2017	Cross-sectional	Sepsis suspected neonates in NICU	Blood	Infection	Disk diffusion	303	16 (18.2)	3 (18.8)
Kejela et al., 2022 [48]	Oromia	Mettu	November 2019 to April 2020	Cross-sectional	Inpatients	Wound swab, nasal swab	Infection	Dilution/MIC	384	126 (32.8)	10 (7.9)
Daka D, 2014 [49]	Sidama	Hawassa	August 2013 to December 2014	Cross-sectional	Health care workers	Hand swab	Carriage	Disk diffusion	152	84 (55.3)	34 (40.4)
Guta et al., 2014 [50]	Sidama	Hawassa	November 2010 to June 2011	Cross-sectional	Patients with infected surgical wounds	Infected surgical wound swab	Infection	Disk diffusion	100	45 (45)	30 (66.7)
Deyno et al., 2017 [51]	Sidama	Hawassa	February to November 2016	Cross-sectional	Out patients with ear infection	Ear swab	Infection	Disk diffusion	117	33 (28.2)	25 (75.8)
Mechal et al., 2021 [52]	Sidama	Hawassa	October 2018 to February 2019	Cross-sectional	Adult outpatients (≥ 18 years)	Urine	Infection	Disk diffusion	387	21 (16.5)	2 (9.5)
Abebe T et al., 2023 [53]	Somali	Jigjiga	May 1 to June 30, 2020	Cross-sectional	External ocular infection suspects	Conjunctival swab	Infection	Disk diffusion	288	95 (60.5)	4 (4.2)
Wasihun et al., 2015 [54]	Tigray	Mekelle	March to October 2014	Cross-sectional	Febrile patients	Blood	Infection	Dilution/MIC	514	54 (10.5)	1 (1.8)

### Prevalence of VRSA in Ethiopia

**Fig. 2:**
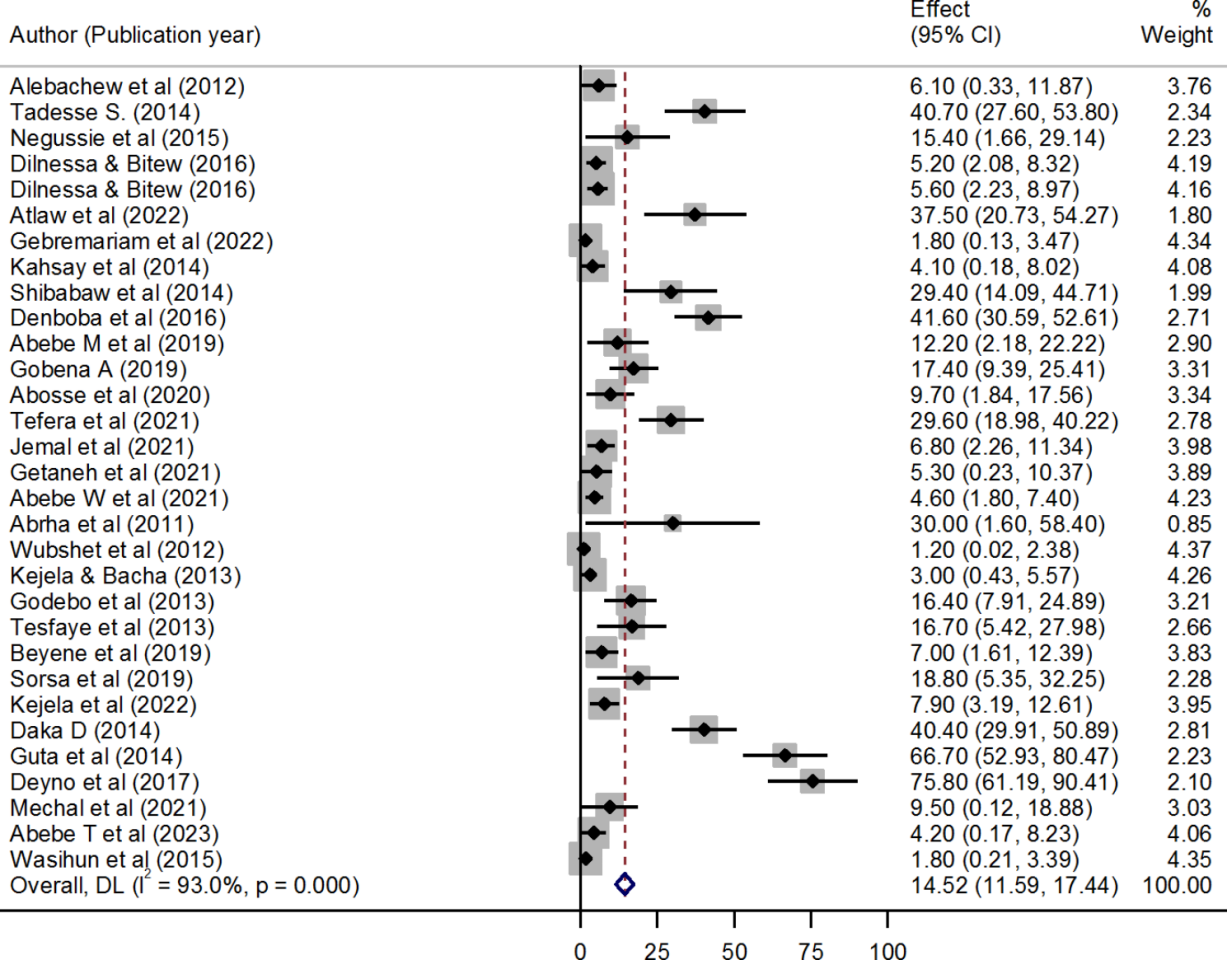
Forest plot showing the pooled prevalence of VRSA in Ethiopia from random-effect model analysis

In this systematic review and meta-analysis, the overall pooled prevalence of VRSA in Ethiopia was 14.52% (95% CI: 11.59, 17.44). A huge discrepancy in the prevalence of VRSA was revealed among the included studies, ranging from 1.2% (95% CI: 0.02, 2.38) reported in Jimma to 75.8% (95% CI: 61.19, 90.41) reported in Hawassa. Significantly high level of heterogeneity was observed among studies (I^2^ = 93.0%, p < 0.001) (Fig. 2).

### Subgroup analysis of VRSA prevalence in Ethiopia

Subgroup analysis was carried out based on region, city, year of publication, study design and AST method. The region-based subgroup analysis depicted highest pooled prevalence of 47.74% (95% CI: 17.79, 77.69) in Sidama region, followed by 14.82% (95% CI: 8.68, 19.88) in Amhara region, while Oromia region had the least pooled prevalence 8.07% (95% CI: 4.09, 12.06). High heterogeneity was demonstrated in all included regions of the country. The pooled prevalence of VRSA was highest in Hawassa 47.74% (95% CI: 17.79, 77.69), followed by 36.71% (95% CI: 24.99, 48.43) in Dessie. Relatively low level of heterogeneity was observed from studies conducted in Dessie (I^2^ = 37.8%, p = 0.205) and Bahir Dar (I^2^ = 44.7%, p = 0.179), whereas no heterogeneity (I^2^ = 0.0%, p = 0.721) was seen among studies in Gondar. Nevertheless, there was high heterogeneity in Addis Ababa, Debre Markos, Hawassa and Jimma. Likewise, highest pooled prevalence of VRSA 21.01% (95% CI: 11.58, 30.45) was observed in the years 2015–2017, and low level of heterogeneity was seen among studies in the period 2018–2020. The prevalence of VRSA pooled from studies showed increment from the period ≤ 2014 to 2015–2017, then declined in the later publication years. The subgroup analysis based on AST methods depicted a significant variation in pooled prevalence of VRSA (6.3% (95% CI: 3.14, 9.43) for MIC-based methods, and 18.4% (95% CI: 14.03, 22.79) for disk diffusion AST method). On the other hand, the prevalence of VRSA in terms of study design was 15.07% (95% CI: 11.82, 18.31) in cross-sectional studies and 12.54% (95% CI: 4.66, 20.42) in studies with retrospective design (Table 2).

**Table 2:** Subgroup analysis of VRSA by region, city, publication year and AST method

Subgroups	Category	No of studies	No of S. aureus isolates tested, N	Pooled prevalence of VRSA, N (%)	95% CI	Heterogeneity test (I^2^)	P-value	Heterogeneity between groups (p-value)
Region	Central	7	592	61 (11.25)	(5.78, 16.72)	89.4%	< 0.001	0.027
	Amhara	10	821	111 (14.28)	(8.68, 19.88)	88.2%	< 0.001	
	Oromia	8	603	47 (8.07)	(4.09, 12.06)	81.7%	< 0.001	
	Sidama	4	183	91 (47.74)	(17.79, 77.69)	96.2%	< 0.001	
	Total pooled	29	2199	310 (16.14)	(12.78, 19.50)	93.3%	< 0.001	
City	Addis Ababa	7	592	61 (11.25)	(5.78, 16.72)	89.4%	< 0.001	< 0.001
	Debre Markos	3	185	29 (14.71)	(0.11, 29.54)	90.2%	< 0.001	
	Dessie	2	111	42 (36.71)	(24.99, 48.43)	37.8%	0.205	
	Bahir Dar	2	117	18 (13.51)	(5.96, 21.05)	44.7%	0.179	
	Gondar	3	408	22 (5.22)	(3.07, 7.38)	0.0%	0.721	
	Jimma	6	461	34 (7.01)	(2.71, 11.31)	81.4%	< 0.001	
	Hawassa	4	183	91 (47.74)	(17.79, 77.69)	96.2%	< 0.001	
	Total pooled	27	2057	297 (16.50)	(12.98, 20.01)	93.7%	< 0.001	
Publication year	≤ 2014	11	731	131 (20.30)	(13.43, 27.17)	96.1%	< 0.001	0.005
	2015–2017	6	550	80 (21.01)	(11.58, 30.45)	96.6%	0.001	
	2018–2020	5	260	32 (11.69)	(7.15, 16.22)	33.5%	0.198	
	≥ 2021	9	807	72 (8.27)	(4.54, 11.99)	84.1%	< 0.001	
	Total pooled	31	2348	315 (14.52)	(11.59, 17.44)	93.0%	< 0.001	
Study design	Cross-sectional	26	1822	256 (15.07)	(11.82, 18.31)	93.3%	< 0.001	0.561
	Retrospective	5	526	59 (12.54)	(4.66, 20.42)	90.5%	< 0.001	
	Total pooled	31	2348	315 (14.52)	(11.59, 17.44)	93.0%	< 0.001	
Specimen type	Wound swab	7	538	83 (17.97)	(8.72, 27.21)	93.7%	< 0.001	0.184
	Blood	7	512	42 (8.62)	(4.16, 13.09)	79.9%	< 0.001	
	Nasal swab	2	203	15 (15.08)	(-10.70, 40.85)	91.0%	0.001	
	Ear discharge	3	185	61 (40.45)	(0.06, 80.83)	98.0%	< 0.001	
	Conjunctival swab	2	137	11 (9.29)	(-2.74, 21.33)	76.1%	0.041	
	Multiple samples	7	636	55 (6.84)	(3.30, 10.38)	88.5%	< 0.001	
	Total pooled	28	2211	267 (13.08)	(10.22, 15.95)	92.6%	< 0.001	
AST method	MIC-based	7	719	58 (6.29)	(3.14, 9.43)	84.9%	< 0.001	< 0.001
	Disk diffusion	24	1629	257 (18.41)	(14.03, 22.79)	94.0%	< 0.001	
	Total pooled	31	2348	315 (14.52)	(11.59, 17.44)	93.0%	< 0.001	

### Meta-regression

**Fig. 3:**
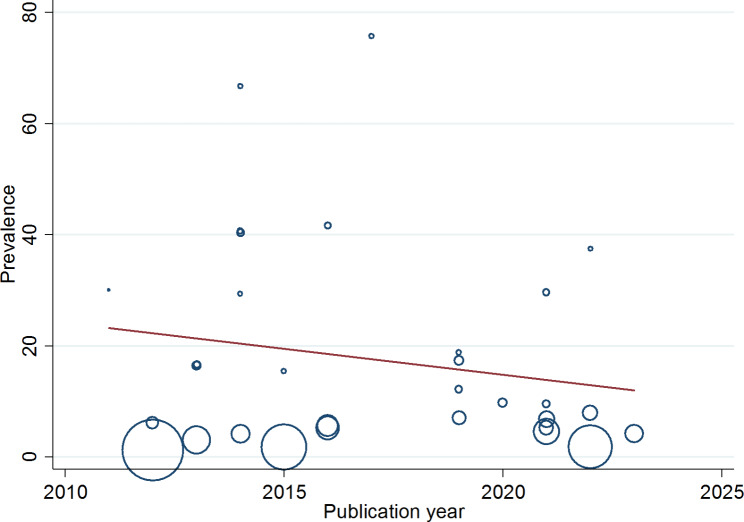
Meta-regression analysis of VRSA infections based on publication years

Meta-regression was carried out to further explore the potential sources of heterogeneity or variability among studies included in the meta-analysis. We included continuous study characteristics as covariates including publication year, sample size and total number of *S. aureus* isolates in the meta-regression model and assess their potential influence on the overall effect size (pooled prevalence of VRSA) (Fig. 3). In this study, total number of *S. aureus* isolates was found to be the responsible variable for the existence of heterogeneity among studies (p = 0.033) (Table 3).

**Table 3:** Meta-regression analysis of prevalence of VRSA by different categories of studies included in the systematic review and meta-analysis

Moderator	No. of studies	Exp(b)	SE	t	P	95% CI
Publication year	31	0.39	0.36	-1.01	0.322	(0.06, 2.62)
Sample size	31	0.99	0.01	-1.53	0.137	(0.98, 1.00)
Total *S. aureus* isolates	31	0.87	0.05	-2.24	0.033*	(0.77, 0.99)

*= Significant causes of heterogeneity

### Publication bias

**Fig. 4:**
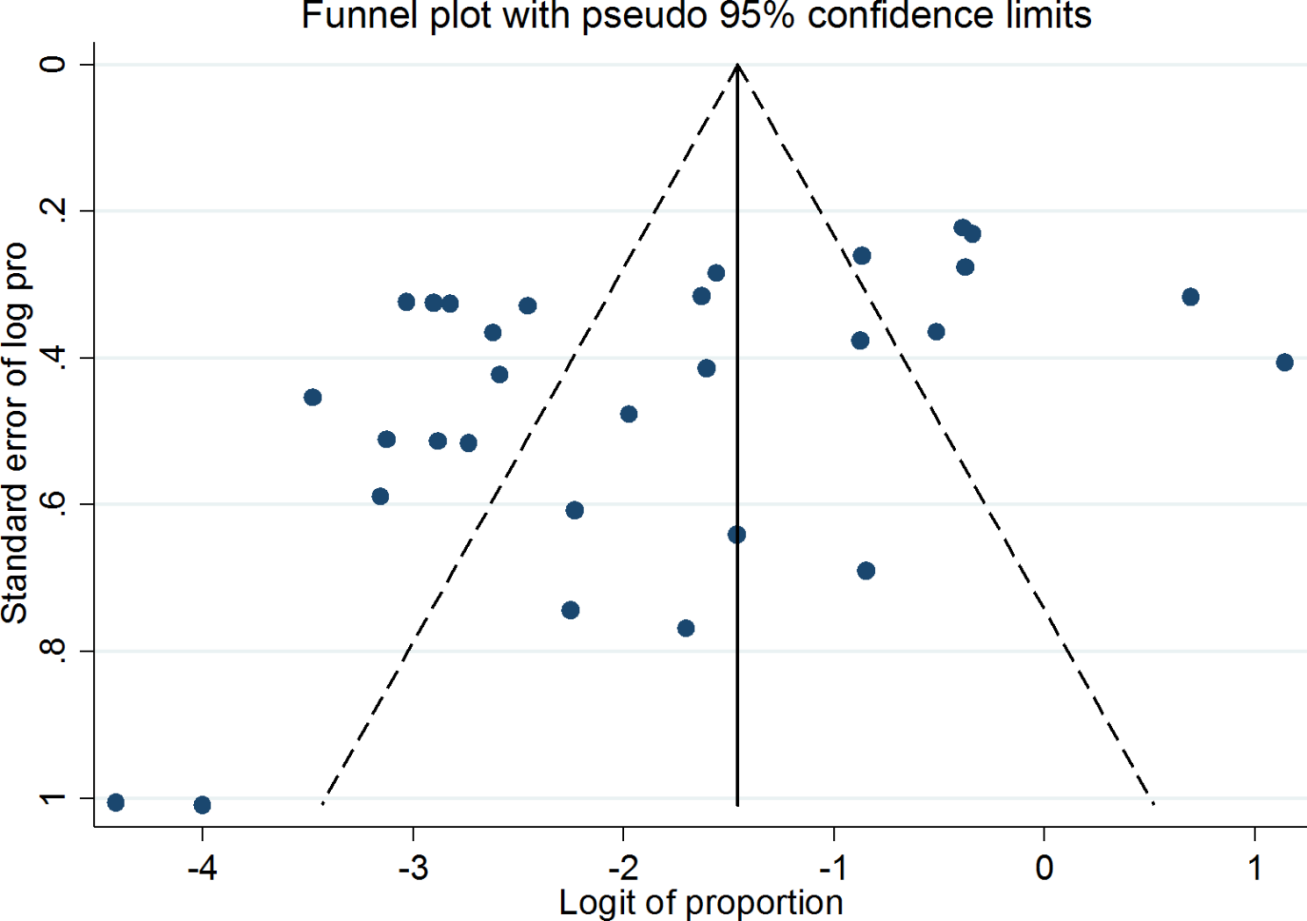
Funnel plot on the prevalence of VRSA in Ethiopia illustrating the presence of publication bias

**Fig. 5:**
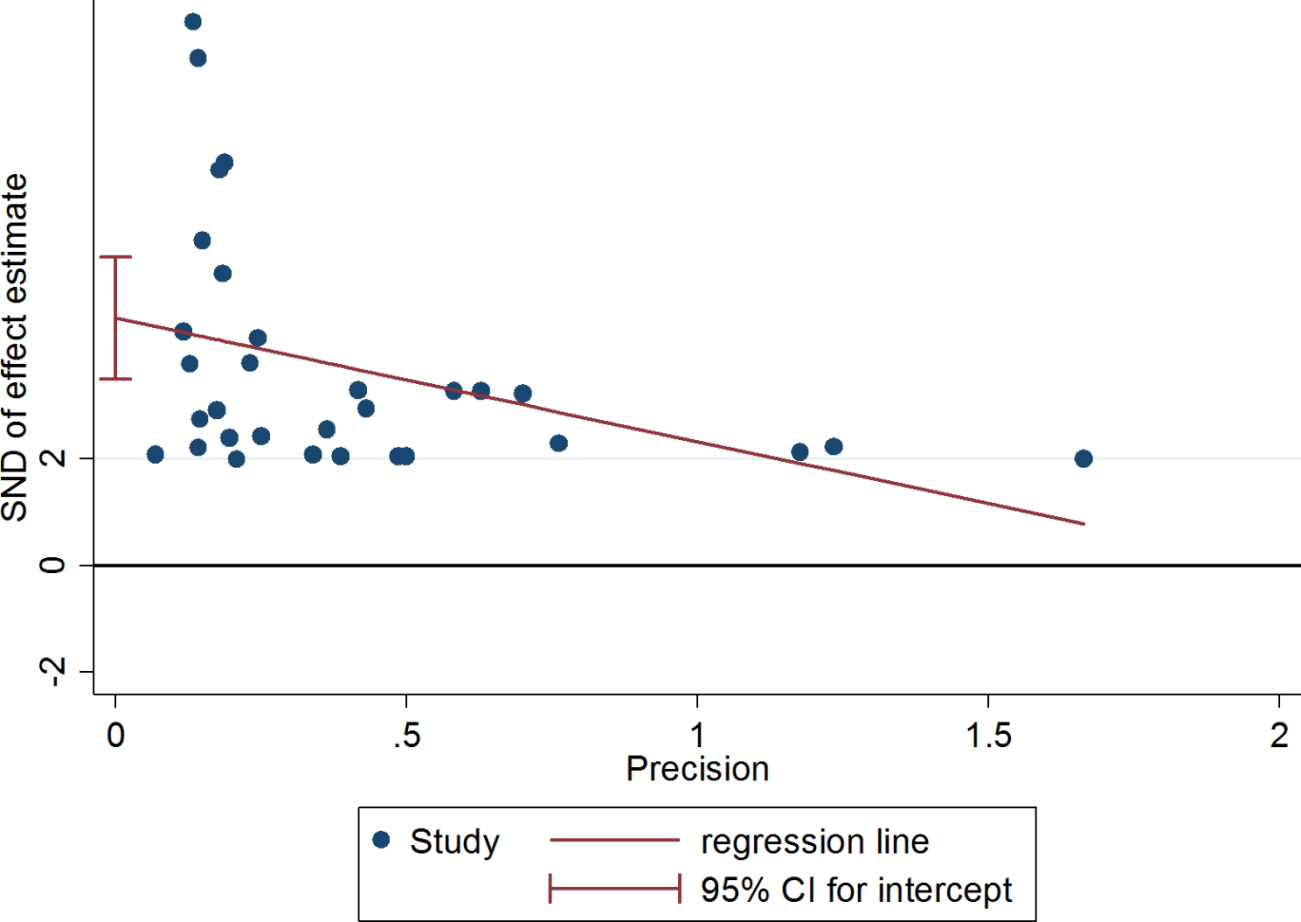
Egger’s test graph depicting publication bias

In this study, the symmetry of the funnel plot illustrated the presence of publication bias, with over 67% of the studies skewed to the left side of the triangular zone (Fig. 4). This finding was further supported by the Egger’s test, which revealed the presence of substantial publication bias (p < 0.001) (Table 4) (Fig. 5).

**Table 4:** Egger’s test statistics of the prevalence of VRSA in Ethiopia

Std-Eff	Coef.	Std. Err.	t	P	95% CI
Slope	-2.31	1.03	-2.25	0.032	-4.41, -0.21
Bias	4.63	0.56	8.25	< 0.001	3.48, 5.77

### Trim and fill analysis of pooled prevalence of VRSA in Ethiopia

Attributable to the presence of marginally significant publication bias, we performed a trim and fill analysis. After incorporating 16 additional studies, the trim and fill analysis revealed a pooled prevalence of 3.56% (95% CI: 0.39, 6.73) VRSA in Ethiopia (Table 5).

**Table 5:** Trim and fill analysis of the prevalence of VRSA in Ethiopia

Method	Pooled est.	95% CI		Asymptotic		No. of studies
		**Lower**	**Upper**	**z-value**	**p-value**	
Fixed	3.966	3.322	4.611	12.062	< 0.001	31
Random	14.516	11.593	17.438	9.734	< 0.001	
Test for heterogeneity: Q = 426.039 on 30 degrees of freedom (p < 0.001)						
Moment-based estimate of between studies variance = 50.509						
Trimming estimator: Linear						
Meta-analysis type: Fixed-effects model						
Iteration	**Estimate**	**Tn**	**# To trim**		**Diff**	
1	3.966	462	14		496	
2	2.825	485	16		46	
3	2.735	485	16		0	
Filled						
Meta-analysis						
Method	**Pooled est.**	**95% CI**		**Asymptotic**		**No. of studies**
		**Lower**	**Upper**	**z-value**	**p-value**	
Fixed	2.735	2.107	3.364	8.533	< 0.001	47
Random	3.561	0.391	6.730	2.202	0.028	
Test for heterogeneity: Q = 848.753 on 46 degrees of freedom (p < 0.001)						
Moment-based estimate of between studies variance = 95.679						

### Sensitivity analysis

Based on the results of the sensitivity analysis, which was conducted using a random effect model, the pooled effect size fell within the 95% CI of the overall pooled affect size when the individual studies were omitted. This demonstrated that no single study had an impact on the overall pooled prevalence of VRSA infection in Ethiopia (Table 6).

**Table 6:** Sensitivity analysis of the included studies

S No.	Study omitted	Estimate	95% CI
1	Alebachew et al., 2012 (24)	14.89	11.89, 17.89
2	Tadesse S., 2014 (25)	13.72	10.86, 16.59
3	Negussie et al., 2015 (26)	14.49	11.54, 17.45
4	Dilnessa et al., 2016 (27)	15.07	12.01, 18.12
5	Dilnessa & Bitew, 2016 (28)	15.02	11.98, 18.07
6	Atlaw et al., 2022 (29)	14.01	11.11, 16.92
7	Gebremariam et al., 2022 (30)	15.51	12.30, 18.72
8	Kahsay et al., 2014 (31)	15.05	12.02, 18.08
9	Shibabaw et al., 2014 (32)	14.16	11.23, 17.09
10	Denboba et al., 2016 (33)	13.49	10.66, 16.32
11	Abebe M et al., 2019 (34)	14.59	11.62, 17.56
12	Gobena A, 2019 (35)	14.37	11.42, 17.33
13	Abosse et al., 2020 (36)	14.69	11.72, 17.68
14	Tefera et al., 2021 (37)	13.96	11.05, 16.86
15	Jemal et al., 2021 (38)	14.89	11.89, 17.91
16	Getaneh et al., 2021 (39)	14.95	11.94, 17.96
17	Abebe W et al., 2021 (40)	15.13	12.06, 18.21
18	Abrha et al., 2011 (41)	14.37	11.44, 17.30
19	Wubshet et al., 2012 (42)	15.70	12.40, 19.00
20	Kejela & Bacha, 2013 (43)	15.23	12.14, 18.32
21	Godebo et al., 2013 (44)	14.43	11.47, 17.38
22	Tesfaye et al., 2013 (45)	14.44	11.49, 17.40
23	Beyene et al., 2019 (46)	14.86	11.86, 17.86
24	Sorsa et al., 2019 (47)	14.40	11.45, 17.35
25	Kejela et al., 2022 (48)	14.84	11.83, 17.85
26	Daka D, 2014 (49)	13.49	10.65, 16.32
27	Guta et al., 2014 (50)	12.85	10.13, 15.57
28	Deyno et al., 2017 (51)	12.65	9.98, 15.31
29	Mechal et al., 2021 (52)	14.69	11.71, 17.66
30	Abebe T et al., 2023 (53)	15.04	12.01, 18.07
31	Wasihun et al., 2015 (54)	15.55	12.32, 18.77
	**Combined**	**14.51**	**11.59, 17.44**

## Discussion

Nowadays, frequent use of vancomycin as the drug of choice for treatment of infections caused by MRSA and other Gram-positive MDR pathogens has led to the emergence of *S. aureus* isolates with high resistance to vancomycin [13, 55, 56]. According to our evidence so far, we carried out the first large-scale systematic review and meta-analysis of available data on the epidemiology of VRSA in Ethiopia. The main aim of this study was to determine the national pooled prevalence of VRSA in Ethiopia by pooling data from various studies and assess the distribution patterns of VRSA across the country. The overall pooled prevalence estimate of VRSA in Ethiopia was found to be 14.52% (95% CI: 11.59, 17.44), with high level of heterogeneity (I^2^ = 93.0%, p < 0.001). This finding is comparable with a previous review reporting the pooled prevalence of VRSA in Africa 16% (95% CI: 3, 35) [15]. On the contrary, the finding of the present systematic review and meta-analysis is massively higher than global studies that reported an overall pooled prevalence of VRSA as 1.5% (95% CI: 1.0, 2.0] [16] and 6% (95% CI: (0.04, 0.09) [15]. In addition, our overall pooled prevalence finding is higher than a systematic review and meta-analysis studies conducted in Iran which only reported 24 VRSA isolates from the included thirteen studies with a pooled prevalence of 2.4% [57] and in the Middle east which reported a total of only 19 VRSA isolates with a pooled prevalence of 2.1% [58]. This higher finding indicates the huge burden of VRSA in Africa including Ethiopia than other continents as evidenced by lower findings reported from Asia 5% (95% CI: 0.03, 0.08), South America 3% (95% CI: 0.00, 0.17), North America 4% (95% CI: 0.02, 0.07), and Europe 1% (95% CI: 0.00, 0.05) [15]. The possible reasons for the higher rate of VRSA could possibly be poor hygiene standards [59], inadequate monitoring of nosocomial infections, and improper use of available antibacterial drugs in Africa in comparison to developed countries [60]. Furthermore, the problem will probably get worse as a result of the irrational use of antibiotics in health facilities and the accessibility of antibacterial drugs over the counter in many developing countries [61]. Nevertheless, our findings indicated a higher prevalence of VRSA strains within the country, revealing a more concerning level of *S. aureus* resistance to vancomycin than initially estimated or anticipated. The discrepancy in these estimations could be attributed to several factors. Firstly, the absence of a molecular approach for vancomycin resistance detection in almost all studies conducted in Ethiopia has contributed to an inadequate global report. Additionally, the absence of a national genomic repository in the country further complicates the situation. Moreover, a significant number of these studies did not adhere to specific guidelines, such as the recommendations provided by the Centers for Disease Control and Prevention, resulting in incomplete adherence to standardized protocols [62].

This significantly high pooled prevalence of VRSA in Ethiopia is indicative of the alarming widespread of multidrug-resistant *S. aureus* throughout all regions of the country. This finding, compounded with an escalated reports of MRSA in the country 10.94% [63], 32.5% [64], 47% [17] and 50.0% [65], necessitate urgent improvements to the national treatment guidelines to incorporate alternative, highly effective antimicrobial agents targeting MRSA. Simultaneously, the implementation of comprehensive antimicrobial stewardship strategies, accompanied by robust systemic surveillance, is imperative. Additionally, to curb the transmission of VRSA, it is essential to prioritize infection control measures such as contact precautions, meticulous screening, proper sterilization of healthcare equipment, and ensuring a sanitized environment [66].

Besides, this study revealed high level of heterogeneity (I^2^ = 93.0%, p < 0.001) depicting the presence of variations among included studies. The likely reason for this immense heterogeneity could be variations in methodology, study participants, study design and sample size all of which exert an influence on the prevalence of VRSA. A key contributor to this heterogeneity is the diversity of the target population, encompassing a range of individuals such as healthy food handlers, wound patients, children, healthcare professionals, burn patients, and individuals with diverse underlying medical conditions. Notably, surgical wound and burn patients are particularly prone to staphylococcal infections due to the loss of their skin’s protective barrier and the immunosuppression resulting from the systemic inflammatory response induced by the damaged tissue. This variety stemming from the diverse target population undoubtedly contributes to the elevated level of heterogeneity observed in this study.

Due to the diverse nature of the included studies, we anticipated heterogeneity and considered subgroup analysis in terms of region, city, publication year, study design, specimen type and AST method. In the subgroup analysis, we reported a moderate increment in the pooled prevalence of VRSA from the period ≤ 2014 (20.30%) to 2015–2017 (21.01%). This finding is in line with previous report of global meta-analysis, which reported a rise in the pooled prevalence of VRSA from the period < 2006 (2%), 2006–2014 (5%), 2015–2020 (7%) [15]. In addition, similar finding was revealed in a global study depicting a twofold upsurge in pooled prevalence of VRSA from 1.2% in studies conducted before 2010 to 2.4% in studies conducted after 2010 [16]. Nevertheless, our finding revealed a decline in pooled prevalence in the latest publication years. The reason for such discrepancy could be due to variations in the number of included studies across the categorized years. The recent subgroup periods 2018–2020 and ≥ 2021 comprised of fewer number of studies, which could be due to a shift in healthcare priorities to SARS-CoV-2 (COVID-19) pandemic response, and thus the number of studies may have decreased in these periods, causing the findings in these periods to be underestimated.

Region-based pooled prevalence was also estimated. The highest pooled prevalence of 47.74% (95% CI: 17.79, 77.69) was depicted in Sidama region, which is about three-times higher than Amhara region 14.82% (95% CI: 8.68, 19.88), four-times higher than the central (Addis Ababa) region 11.25% (955CI: 5.78, 16.72), and six-times higher than the least pooled prevalence from Oromia region 8.07% (95% CI: 4.09, 12.06). This regional variation could be attributable to differences in the study population, study period and antimicrobial susceptibility testing method and type of clinical sample used to isolate VRSA. Although such highest pooled prevalence in some regions and cities were mainly attributed to the use of disk diffusion technique of VRSA detection, the magnitude is still high and need further evaluation and genomic confirmation.

In the accurate diagnosis of VRSA, the role of clinical laboratory is critical for detecting, isolating and determining the antimicrobial susceptibility pattern [67]. In this regard, various techniques can be used to determine the resistance or susceptibility of *S. aureus* against vancomycin. In this study, the VRSA rates were significantly different based on AST methods. The pooled prevalence of VRSA using disk diffusion AST method (18.41%) is higher than the MIC-based methods (6.3%). This finding clearly showed that disk diffusion AST method overestimates the VRSA prevalence. Disk diffusion technique is not a reliable method as it showed poor sensitivity in differentiating the wild type isolates from isolates with non-*vanA*-inferred glycopeptide resistance [68, 69]. The MIC test technique of detecting vancomycin resistance, which include E-test and broth dilution tests, is considered a gold standard technique. However, these methods are not commonly being used in clinical laboratories of developing countries due to the fact that they are time-consuming, costly, labor intensive, and technically difficult. Consequently, clinical laboratories in developing countries are still using disk diffusion method to detect VRSA and this might result in overestimation of VRSA. Despite the incredibly high overall prevalence of 14.52% from all pooled studies, the pooled prevalence from studies using correct VRSA detection methods (MIC-based methods) was 6.3%, which is still high and a cause of national concern. This finding showed that there is an urgent need to improve the methods to determine vancomycin resistance in Ethiopia, and efforts should be directed at improving this nationally. In this sense, the incorporation of MIC-based methods for VRSA detection in routine clinical laboratory tests in Ethiopia is of paramount significance to show the real burden, and should be given due attention.

The methodology employed in a study plays a crucial role in accurately assessing the burden of a pathogen. Our meta-regression analysis identified the total number of *S. aureus* isolates as a significant factor contributing to heterogeneity among studies (p = 0.033) while publication year was not found to be a significant cause. It is common for prevalence studies conducted in developing countries, including Ethiopia, to involve a limited number of study participants, primarily due to financial and funding constraints. Consequently, this leads to a small number of bacterial isolates and may contribute to the observed heterogeneity among studies [70]. However, this finding contradicts a global report [16] that identified publication year as a source of heterogeneity.

One of the notable strengths of this study is its comprehensive nature, being the first of its kind to conduct a thorough analysis of VRSA within Ethiopia. It encompasses a wide range of studies conducted across multiple regions and cities of the country, providing a robust overview. Furthermore, the study included various studies done in different target populations using diversified clinical specimens in order to show the clear picture of VRSA in the country. However, the results should be interpreted with caution as the reviewed studies were highly heterogeneous in terms of VRSA magnitude, study setups, study participants, outcomes, diseases conditions, clinical specimens, sample sizes and AST methods, which collectively might introduce bias and have effect on result interpretation. Therefore, to account for this heterogeneity, the random-effects model of DerSimonian and Laird analysis was implemented in the meta-analysis. However, it should be taken into consideration that the DerSimonian-Laird (DSL) estimation method may have limitations when applied to estimate prevalence in studies with small sample sizes, and have shortcomings of being influenced by the number of included studies for meta-analysis and heavily biased when it is applied to proportions [71]. Moreover, subgroup analyses, sensitivity analysis, and meta-regression were conducted to further address and mitigate the impact of heterogeneity on the findings.

## Conclusions

This systematic review and meta-analysis showed an alarmingly high pooled prevalence of VRSA which raises significant concerns for public health. The high burden of VRSA emphasizes the urgency of implementing routine screening practices and ensuring the appropriate utilization of antibiotics for effective management of MRSA infections. Mainly attributable to the overestimation of VRSA burden while using disk diffusion method, there is an urgent need to improve the methods to determine vancomycin resistance in Ethiopia and incorporate MIC-based VRSA detection methods in routine clinical laboratory tests, and efforts should be directed at improving it nationally. Furthermore, it serves as a clear call to action for the development and implementation of robust infection prevention measures and antimicrobial stewardship programs aimed at curbing the emergence and spread of drug resistance in Staphylococcal infections.

### Electronic supplementary material

Below is the link to the electronic supplementary material.

Supplementary Material 1

Supplementary Material 2

## Data Availability

All relevant data are included in the manuscript and its supplementary data.

## Abbreviations

AMR: Antimicrobial resistance
CI: Confidence interval
CLSI: Clinical Laboratory Standards Institute
CSF: Cerebrospinal fluid
MDR: Multidrug resistance
MIC: Minimum inhibitory concentration
MRSA: Methicillin-resistant *Staphylococcus aureus*
NICU: Neonatal and intensive care unit
PRISMA: Preferred Reporting Items for Systematic Reviews and Meta-Analyses
Stata: Statistics and data
VRSA: Vancomycin-resistant *Staphylococcus aureus*
WHO: World Health Organization
