# Supplementary material for: The prevalence of vancomycin-resistant Staphylococcus aureus in Ethiopia: a systematic review and meta-analysis
Source: Antimicrob Resist Infect Control. 2023 Aug 30;12:86. doi: 10.1186/s13756-023-01291-3 (PMC10468870; doi:10.1186/s13756-023-01291-3)
Supplement: Supplementary file 1 — Supplementary Material 1 [file 13756_2023_1291_MOESM1_ESM.docx]

**Table a: Search strategy used to retrieve eligible studies.**

| **MeSH Heading (Key word)** | **Entry terms (synonym)** | **Combination** | **Number of articles** | **Last searching date** | **Electronic database** |
| --- | --- | --- | --- | --- | --- |
| *Staphylococcus aureus* | *S. aureus* | (((((“Staphylococcus aureus”) OR (“S. aureus”)) OR (“Vancomycin resistant Staphylococcus aureus”)) OR (“Vancomycin resistant S. aureus”)) OR (VRSA)) AND (Ethiopia) | 487 | May 5, 2023 | PubMed |
| Vancomycin Resistant *Staphylococcus aureus* | Vancomycin  Resistant *S. aureus* |  |  |  |  |
|  | VRSA |  |  |  |  |
| Ethiopia |  |  |  |  |  |
|  |  | (((((“Staphylococcus aureus”) OR (“S. aureus”)) OR (“Vancomycin resistant Staphylococcus aureus”)) OR (“Vancomycin resistant S. aureus”)) OR (VRSA)) AND (Ethiopia) | 135 | May 5, 2023 | EMBASE |
|  |  | (TITLE-ABS-KEY (“*Staphylococcus aureus*” OR “*S. aureus*” OR “Vancomycin Resistant *Staphylococcus aureus*” OR “Vancomycin Resistant *S. aureus*” OR VRSA) AND TITLE-ABS-KEY (Ethiopia) | 102 | May 12, 2023 | Scopus |
|  |  | “Staphylococcus aureus” and Ethiopia; “S. aureus” and Ethiopia; “Vancomycin Resistant Staphylococcus” and Ethiopia; “Vancomycin Resistant *S. aureus”* and Ethiopia; VRSA and Ethiopia | 11 | May 18, 2023 | Other sources |

**Table b: University repositories and Google search lines used in article search.**

| **S No.** | **Name of University repositories used** | **Address** |
| --- | --- | --- |
| 1. | Addis Ababa University Institutional repository/Electronic Thesis and Dissertation | <http://www.aau.edu.et/library/resources/aau-institutional-repositoryelectronic-thesis-and-dissertation/> |
| 2. | Bahir Dar University Institutional repository | <http://repository.iifphc.org/handle/123456789/1098> |
| 3. | University of Gondar thesis and dissertation repository | <http://repository.iifphc.org/handle/123456789/1140> |
| 4. | Jimma University open access institutional repository | <https://repository.ju.edu.et/> |
| 5. | Haramaya University Institutional repository | <http://ir.haramaya.edu.et/hru/> |
|  |  |  |
|  | **Google search line used** | |
|  | Vancomycin Resistant *Staphylococcus aureus* in Ethiopia |  |
|  |  |  |
|  | **Google scholar search line used** | |
|  | “*Staphylococcus aureus*” OR “*S. aureus*” OR “Vancomycin Resistant *Staphylococcus aureus*” OR “Vancomycin  Resistant *S. aureus*” OR VRSA AND Ethiopia |  |
